# Competing Models of Work in Quadrupedal Walking: Center of Mass Work is Insufficient to Explain Stereotypical Gait

**DOI:** 10.3389/fbioe.2022.826336

**Published:** 2022-05-12

**Authors:** Delyle T. Polet, John E. A. Bertram

**Affiliations:** ^1^ Biological Sciences, University of Calgary, Calgary, AB, Canada; ^2^ Cell Biology and Anatomy, Cumming School of Medicine, University of Calgary, Calgary, AB, Canada

**Keywords:** quadrupedal model, trajectory optimization, work minimization, mammal, pendular recovery

## Abstract

The walking gaits of cursorial quadrupedal mammals tend to be highly stereotyped as a four-beat pattern with interspersed periods of double and triple stance, often with double-hump ground reaction force profiles. This pattern has long been associated with high energetic economy, due to low apparent work. However, there are differing ways of approximating the work performed during walking and, consequently, different interpretations of the primary mechanism leading to high economy. A focus on Net Center of Mass (COM) Work led to the claim that quadrupedal walking is efficient because it effectively trades potential and kinetic energy of the COM. Individual Limbs COM Work instead focuses on the ability of the limbs to manage the trajectory of the COM to limit energetic losses to the ground (“collisions”). By focusing on the COM, both these metrics effectively dismiss the importance of rotation of the elongate quadrupedal body. Limb Extension Work considers work required to extend and contract each limb like a strut, and accounts for the work of body pitching. We tested the prescriptive ability of these approximations of work by optimizing them within a quadrupedal model with two approximations of the body as a point-mass or a rigid distributed mass. Perfect potential-kinetic energy exchange of the COM was possible when optimizing Net COM Work, resulting in highly compliant gaits with duty factors close to one, far different than observed mammalian gaits. Optimizing Individual Limbs COM Work resulted in alternating periods of single limb stance. Only the distributed mass model, with Limb Extension Work as the cost, resulted in a solution similar to the stereotypical mammalian gait. These results suggest that maintaining a near-constant limb length, with distributed contacts, are more important mechanisms of economy than either transduction of potential-kinetic energy or COM collision mitigation for quadrupedal walking.

## 1 Introduction

The walking gait of many quadrupedal mammals is highly stereotyped. Hildebrand found that most mammals use a lateral or diagonal sequence gait, where each leg touches down individually in a “four-beat” pattern (Hildebrand, 1976). A minimum of two limbs are always on the ground, interspersed with periods of triple limb stance at transfer of support (and four-limb stance at the slowest gaits). At slow speeds, quadrupedal mammals rarely use “two-beat” gaits, where fore and hind limbs transfer support simultaneously.

Many cursorial mammals exhibit a bimodal or “double hump” ground reaction force profile in walking (Jayes and Alexander, 1978; Bobbert et al., 2007; Farrell et al., 2014; Basu et al., 2019). This has been explained as a work-minimizing strategy in humans (Tucker, 1975), and in canids (Usherwood et al., 2007; Polet and Bertram, 2019). The double-hump profile can be a diagnostic feature of walking in humans (Hubel and Usherwood, 2015) and horses (Biknevicius et al., 2004).

The ubiquity of this gait suggests a common cause, and energetic economy has been offered as a potential explanation. While gait may arise from the organization of spinal circuitry (Guertin, 2013) and periodic responses to sensory cues (e.g*.* Fukuoka et al., 2015), these neuronal controls likely serve some adaptive function themselves. By investigating the extent to which gait emerges from energetics, we can better understand the adaptive context of motor control, and how other biological and mechanical factors fill in the gaps left by an energetic perspective.

Many factors contribute to energetic cost in locomotion, but positive muscular work is likely a primary contributor (van der Zee et al., 2019; Riddick and Kuo, 2020). Muscular work is often impossible to measure and difficult to estimate. While whole body metabolic power can be measured through gas exchange, this requires specialized equipment and long-duration trials. In contrast, kinetic and kinematic measurements can be collected relatively easily over a handful of strides. From these measurements, various measures of work can be calculated, often with the goal of approximating a metric that correlates to muscular work.

The most commonly used metric of work in locomotion is Net Center of Mass Work (Net COM Work, or NCW). This sums the fluctuations in gravitational potential (*E*_*p*_) and kinetic energy (*E*_*k*_) of the center of mass, which are equivalent to Net COM Power:

$$\text{Net COM Work}=\int_{0}^{T}|F_{\text{net}}\cdot V_{\text{COM}}{|}^{+}\text{dt}$$ (1)

$$\equiv \int_{0}^{T}|\dot{E_{p}}+\dot{E_{k}}{|}^{+}\text{dt},$$ (2)

where **F**_net_ is the net ground reaction force (GRF), **V**_COM_ is the center of mass velocity and |⋅|^+^ is the positive part function. This metric can be measured easily in steady gaits with force plates knowing only the average horizontal velocity of the animal. Eq. 2 establishes the connection between NCW and pendular recovery (Cavagna et al., 1977): when pendular recovery is high, NCW is low and vice versa. Likewise, center of mass work has been parameterized in the “collision angle” framework (Lee et al., 2011), comparing the angle between **F**_net_ and **V**_COM_ implied in the dot product of Eq. 1.

One issue with NCW is that it cannot account for simultaneous positive and negative work. During transfer of support, one limb pushes forward, performing positive work, while the other limb pushes backward, performing negative work. These contributions largely cancel out, generating no apparent NCW, but contribute to the total cost of locomotion (Donelan et al., 2002a,b). A simple fix to NCW is the Individual Limbs COM Work (ILCW), which considers each limb’s instantaneous contribution to the center of mass work:

$$\text{ILCW}=\int_{0}^{T}\underset{i}{\sum }|F_{i}\cdot V_{\text{COM}}{|}^{+}\text{dt},$$ (3)

where **F**_*i*_ is the ground reaction force of limb *i*. Due to frequent periods of simultaneous limb contact on the same force platform, separating ground reaction forces for each limb can be difficult in quadrupedal gait. However, use of center of pressure measurements (Jayes and Alexander, 1978), or multiple plates (Bertram et al., 1997) can alleviate these difficulties.

**FIGURE 1:**
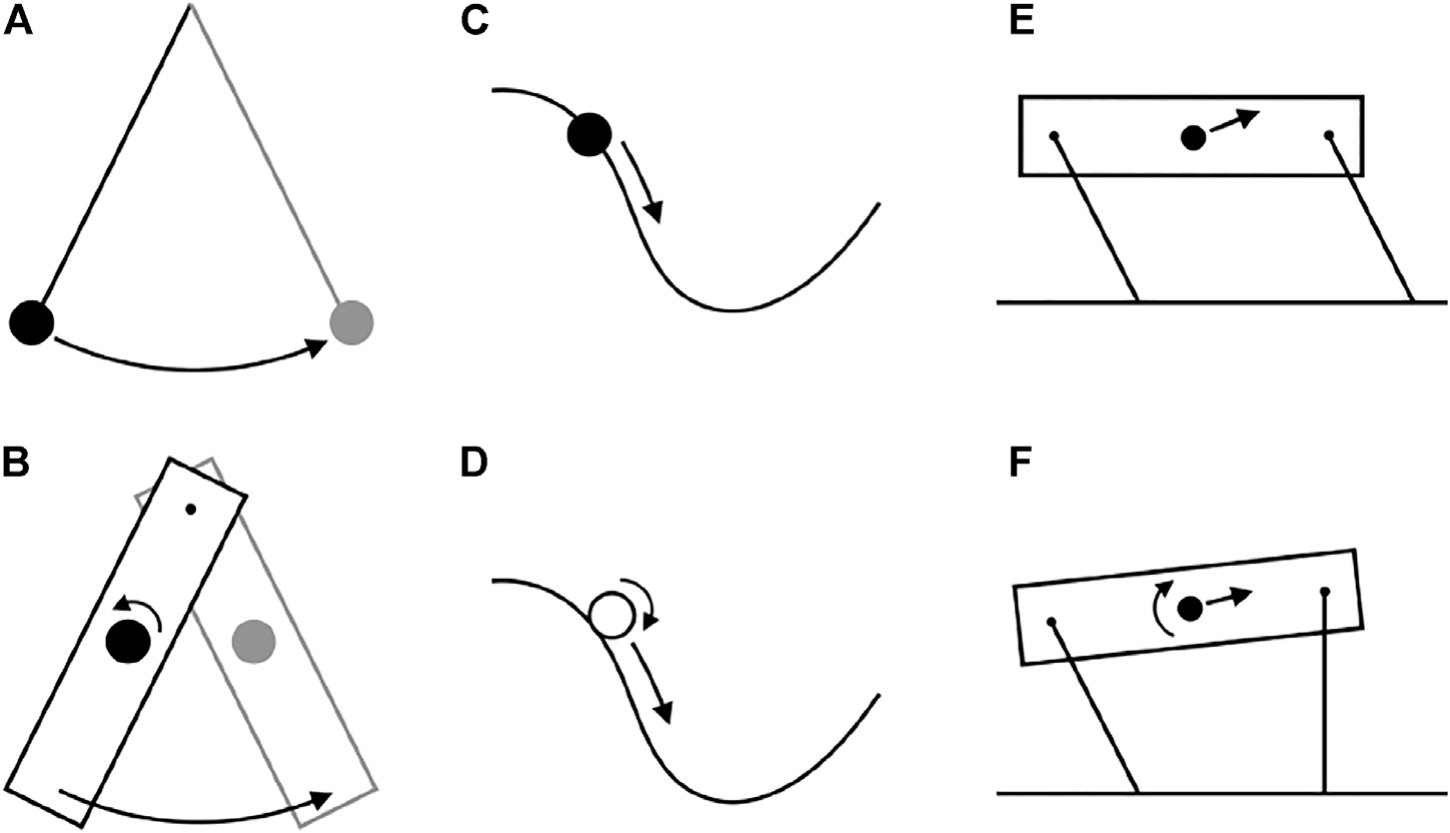
Some examples of conservative systems with differing pendular recovery. Some systems have perfect pendular recovery (top row) while others have low pendular recovery (bottom row) because of energy passively transferred to rotational kinetic energy. **(A)** The classic point mass pendulum exhibits perfect exchange between center of mass kinetic and gravitational potential energy **(B)** A physical pendulum does not. **(C)** A bead on a frictionless wire also has perfect pendular recovery, while **(D)** a ball rolling down a hill does not. **(E)** A quadruped, modelled as a 4-bar linkage with massless legs, can get perfect pendular recovery during vaulting by performing a walking trot. **(F)** The same quadruped gets lower pendular recovery using a four-beat walk.

By focusing on center of mass energetics, these metrics have effectively ignored body rotation. Indeed, some influential models of quadrupedal locomotion assume that effects of rotation are small contributors to cost (Cavagna et al., 1977; Ruina et al., 2005; Lee et al., 2011; Usherwood and Self Davies, 2017). NCW and ILCW can be calculated when the body undergoes extreme rotation, but would be expected to have lower fidelity to muscular work if rotational energies are large. Indeed, Ruina and Bertram (2003) describe how simple passive systems can exhibit finite NCW, when no work is actually performed (Figure 1). In these cases, Net System Work (NSW; total changes in translational and rotational kinetic, and gravitational potential energy) is zero, but NCW increases as gravitational potential and translational kinetic energy convert to rotational kinetic energy.

Another metric can better account for rigid body rotation. Recognizing that ground reaction forces from individual legs are largely aligned with the leg axis of cursorial mammals (Jayes and Alexander, 1978), it is convenient to model the leg as a strut extending and contracting along its axis (Fischer and Blickhan, 2006; Lee et al., 2008). In this framework, one can calculate the Limb Extension Work (LEW):

$$\text{Limb Extension Work}=\int_{0}^{T}\underset{i}{\sum }|F_{i}\cdot {\dot{l}}_{i}{|}^{+}\text{dt},$$ (4)

where *F*_*i*_ is ground reaction force from leg *i* along the leg axis and *l*_*i*_ is the instantaneous length of leg *i*. This has also been called “radial leg work” by some authors (Lee et al., 2008, 2014). This metric is relatively difficult to measure in animals, as it requires not only separate ground reaction forces, but also synchronized kinematic measurement of limb length. There may also be some ambiguity about the appropriate limb “length” to use, due to the complexities of limb attachment (e.g*.* as through the muscular sling of the forelimbs). Finally, it also neglects the work of forces not aligned with the leg axis–either using the limb as a lever (Gray, 1944) or taking advantage of hypothesized leg linkages (Usherwood, 2020b, 2022). However, the axial force assumption remains a useful approximation and simplification (at least for large parasagittal mammals; Fischer and Blickhan, 2006), especially in a modelling context.

All the above metrics have been used in a modelling or prescriptive framework. Cavagna et al. (1977) noticed that the walking gaits of quadrupeds, like bipeds, exhibit out-of-phase kinetic and potential energies of the center of mass. Because the resulting NCW is low (Eq. 2), this “pendular” mode of walking has been put forward as the mechanism of low cost in quadrupedal walking (Cavagna et al., 1977; Full and Koditschek, 1999; Fischer and Lilhe, 2011). Alexander and Jayes (1978) optimized Equation 3 in bipedal and quadrupedal models. Usherwood and Self Davies (2017) used Eq. 3 with a point mass model to predict the limb phase relationships used by slow-moving mammals. Usherwood et al. (2007) used a collisional approximation of Eq. 3 in a model with pitch rotational inertia and enforced vaulting phases matching a 4-bar linkage. Alexander (1980), Polet and Bertram (2019) and Polet (2021) used Eq. 4 to determine whether the walking gaits used by quadrupedal mammals optimized work.

If economy is truly an *objective* of locomotion, and these metrics approximate muscular work well in general for quadrupedal gait, then optimizing the metric should result in a gait similar to the natural one employed. Moreover, to make interpretations about energy saving (or loss mitigating) mechanisms in locomotion, then the simple work metric should ideally be *prescriptive* as well as *descriptive*. The template theory of locomotion (Full and Koditschek, 1999) takes this one step further, positing that the motor control system uses low-order (potentially point-mass) models of the organism to plan behaviour and coordinate muscle activation. From this point of view, low-order models are not merely useful for our understanding of the mechanics of locomotion, but may be used by the organism itself. To what extent can the work metrics above serve as *prescriptive* and *predictive* objectives to be minimized in locomotion?

The “mechanism” of typical quadrupedal walking is important for understanding functional implications. Did the strategy evolve because it stabilizes the organism (Cartmill et al., 2002), or because it is energetically most economical (Hoyt and Taylor, 1981)? If the gait is economical, is that because it efficiently trades kinetic for potential energy (Eq. 2 Cavagna et al., 1977; Griffin et al., 2004; Fischer and Lilhe, 2011), because it produces limb impulses that most effectively manage collisions between the center of mass and the ground (Eq. 3 Bertram, 2016), or because it minimizes aspects of muscle work that are not adequately captured by these heuristics?

To better understand the energetic role of stereotypical quadrupedal walking, we test three competing and increasingly complex work-based cost functions (Eqs 1, 3, 4) under two dynamic models with and without rotational dynamics. We evaluate the ability of these three cost functions to predict duty factors exceeding 0.5, the alternating hind-fore contact pattern, and double-hump ground reaction force profiles typical of walking in cursorial mammals. In forming these reductionist models of locomotion, we prefer if the simpler formulation captures the salient effect of interest- in this case, alternating periods of double and triple limb stance with double-hump ground reaction forces.

## 2 Materials and Methods

### 2.1 Model Dimensions and Empirical Data

The model is two-dimensional in the sagittal plane, and follows the design of Polet and Bertram (2019) and Polet (2021). The body consists of a single rigid trunk, with massless prismatic actuators as legs. The forelimbs attach to the trunk at the shoulder (glenoid) while the hindlimbs attach to the body at the hips (acetabulum). We base the model on an adult Warmblood horse (*Equus ferus caballus*), as a “stereotypical” cursorial quadruped. The maximum hindlimb length (*l*_*H*max_) is set to the empirical length in standing (pes to hips) of 1.38 m. This value was derived from Figure 1 in Bobbert et al. (2007) by scaling to the reported withers height of 1.7 m. The glenoacetabular distance (GAD; hips to shoulders) and maximum forelimb length (manus to shoulders in standing) were set to be equal to the empirical hindlimb length, in order to focus on the effects of the work metrics (rather than complications arising from differences in these lengths). Another model, with more accurate forelimb length and GAD, was also explored using the LEW cost for comparison. In this case, the forelimb length and GAD were measured in the same way as hindlimb length, and found to be 1.14 and 1.63 m respectively.

As the data for this study were recorded at a walking speed of 1.6 m s^−1^, the non-dimensional speed used in the simulation was $U^{\prime }\equiv U/\sqrt{gl_{Hmax}}=0.43$ , where *U* is average horizontal speed, and *g* = 9.81 m s^−2^ is gravitational acceleration. Note that here and elsewhere, we use the prime symbol to denote normalized variables.

The COM is placed along the axis connecting glenoid to acetabulum, at 0.57 times the GAD from the hips, matching the relative impulse produced by the forelimbs by three horses studied by Bobbert et al. (2007). For the distributed mass model, the Murphy number (4 × pitch moment of inertia / body mass / GAD^2^) is set to the empirical value for a dutch warmblood horse of 0.82 (Buchner et al., 1997; Polet, 2021). The point-mass model omitted rotational dynamics but maintained limb length constraints.

Empirical ground reaction forces were extracted from Figure 1 of Bobbert et al. (2007) using WebPlotDigitizer (Rohatgi, 2019). The time between successive touchdowns of the limbs yielded a mean stride time of 1.16 s, and thus a mean stride length *D* of 1.86 m. The stride length normalized to hind limb length was *D*′ = 1.34.

### 2.2 Optimization and Simulation

#### 2.2.1 Problem Specification

Simulations of symmetrical gaits used a contact-invariant method described by Polet and Bertram (2019), simplified to consider only symmetrical gaits as described by Polet (2021). The actuator force for limb *i* (*F*_*i*_) acts along an axis between foot and attachment point to the trunk (hips or shoulders). It is constrained to push only (*F*_*i*_ ≥ 0) and is constrained to be active only when limb length is less than a given value, *via* the complementarity condition

$$F_{i}\left(l_{imax}-l_{i}\right)\geq 0.$$ (5)

Actuator force is instantaneously reflected in ground reaction force, and sliding friction is infinite. Decision parameters include the footfall locations of the left limbs; as the gaits are symmetrical, these are translated by *D*/2 for the right limbs. In the optimization formulation, time is normalized to stride period (*t*′ = *t*/*T*), and simulations occur over the half cycle. States include the kinematics (positions and velocities) of the planar rigid trunk, and the prismatic actuator forces. To enforce gait symmetry, initial kinematics (at *t*′ = 0) are constrained to equal final kinematics (at *t*′ = 0.5), while left-side forces at *t*′ = 0 are constrained to equal their associated right-side forces at *t*′ = 0.5 [e.g. *F*_*LH*_ (0) = *F*_*RH*_(0.5)].

The left-hind (LH) limb is given as the “reference limb” and is constrained to produce zero force at *t* = 0. The right-hind (RH) limb must therefore lift off in the first half cycle (its force must be zero at *t* = 0.5). Either the left-front (LF) or the right-front (RF) limb must touch down in the first half cycle (or always produce zero force); but since the model is planar, either limb can be swapped with no change to the solution dynamics. So, to simplify the formulation, we constrain the left-front limb to touch down in the first half cycle (or always produce zero force). To allow for the left front limb to lift off before touchdown in the first half cycle, we specify left-front actuator force through a trailing (caudal) footfall (*F*_*LFt*_) as well as a leading (rostral) footfall (*F*_*LFl*_) separated by *D*, with *F*_*LFt*_ = 0 at *t*′ = 0.5 and *F*_*LFl*_ = 0 at *t*′ = 0. Consequently, the forces of the right-front limb act through at most one footfall in the first half cycle, and do not have any constraints on force at *t*′ = 0 or 0.5.

A limb exclusion constraint, given as

$$F_{LFt}\int_{0}^{t}F_{LFl}\left(\tau \right)\text{d}\text{τ}=0,$$ (6)

ensured that the forces of the left-front limb acting through the trailing footfall (*F*_*LFT*_) were never active once the forces through the leading footfall (*F*_*LFL*_) exceeded zero. This requires adding $\int_{0}^{t}F_{LFl}\left(\tau \right)d\tau$ as an additional state variable.

Periodicity is enforced by constraining all kinematics (apart from horizontal COM position) to be equal at *t*′ = 0 and *t*′ = 0.5, and constraining left limb forces at *t*′ = 0 to be equal to associated right limb forces at *t*′ = 0.5.

As constraint violation is only evaluated in SNOPT and GPOPS-II at node points, certain constraints could be violated at intermediate points. If the mesh was too sparse, the constraint Eq. 6 could be violated between points, leading to brief periods of five-limb contact. This was fixed by adding an additional complementarity constraint for the simulations minimizing NCW,

$$F_{LFt}{\dot{F}}_{LFl}=0.$$ (7)

Like other complementarity conditions, the path constraints Eqs 5–7 were smoothed using the relaxation parameter method described by Manchester and Kuindersma (2017), which involved augmenting the objective with relaxation parameter and slack variable complementarity penalties.

The predominant cost in the objective is work, using one of Eqs 1, 3, 4. The absolute values for each cost function were transformed using slack variables, as described by Polet and Bertram (2019), following Betts (2010). A force rate penalty of $\sum_{i}\int_{0}^{T}c_{1}{\dot{F}}_{i}^{2}dt$ was added to regularize the cost function and avoid non-smooth force profiles typical of work-minimizing solutions. The scaling factor *c*_1_ was kept at 0.00003, matching Polet (2021) and 100 times smaller than the value used by Polet and Bertram (2019).

In summary, the actuator force rate of change were given as control variables, along with slack variables and relaxation parameters. States were the three planar degrees of freedom of the trunk and its time derivatives, actuator forces and $\int_{0}^{t}F_{LFl}\left(\tau \right)d\tau$ . Decision parameters were the footfall locations of the left limbs. Solutions are constrained to start at a horizontal position 0 and end at *D*/2 in time *T*/2, and hip and shoulders were constrained to remain above ground. The body pitch (relative to the horizontal) was constrained between ± *π*/2. Actuator forces were constrained to be positive (pushing forces with no suction into the ground). All other variables were left with large enough bounds to be effectively unbounded.

#### 2.2.2 Optimization Routine

Optimizations were transcribed using GPOPS-II (v 2.3, Patterson and Rao, 2014) and meshes were refined using the *hp*-adaptive method described by Patterson et al. (2015) and with gradient estimation using central differencing. The resulting nonlinear program was solved using SNOPT (v 7.5, Gill et al., 2005, 2015).

Initial random guesses were created according to the method described in Polet and Bertram (2019). 250 random initial guesses were used per test case. From these guesses, an initial optimization was performed using low relaxation parameter penalties, and a maximum of 500 SNOPT iterations and two mesh iterations. The output from this optimization was downsampled to 16 evenly-spaced grid points, and served as the input to the next round of optimization. In this round, relaxation parameter penalties were increased tenfold, and up to 1000 SNOPT iterations and three mesh iterations were allowed. If mesh tolerances were not satisfied after the three mesh iterations were used, the output was downsampled again and used as a guess for a final round of optimization with up to eight mesh iterations.

If the solution satisfied mesh tolerance, and SNOPT indicated successful convergence, the solution was subjected to an additional mesh refinement step, which interpolated additional collocation points midway between existing points. This refined mesh served as the input guess for an additional round of optimization.

These final solutions were then evaluated for mesh error below tolerance, satisfactory convergence with SNOPT, and satisfaction of complementarity conditions at grid points. Any solutions that did not satisfy these criteria were rejected. Of the remaining solutions, the one that minimized the total objective (work, force rate penalty, and relaxation parameter penalties) overall was selected as the “pseudoglobal” optimum.

## 3 Results

### 3.1 Ground Reaction Force Traces

**FIGURE 2:**
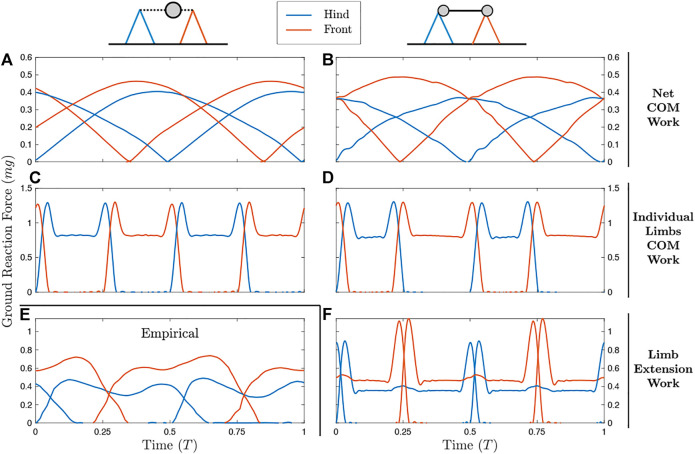
Ground reaction forces (GRFs) for the point mass model (left column) and distributed mass model (right column) with different measures of work (rows). Diagrams show approximate placement of point mass (left) and radii of gyration (right). **(A,B)** Optimizing on Net COM Work results in duty factors close to one and single-peak GRFs. **(C,D)** The Individual Limbs COM Work objective results in alternating periods of single-stance vaulting, with double-hump GRFs. **(E)** Empirically, horses in a medium walk display double-hump GRFs with alternating periods of double and triple support. These features are reproduced only in **(F)** the distributed mass model with Limb Extension Work as the objective. For the planar model results, left and right limb contact can be swapped with no change to the model results, and so only hind and forelimbs are distinguished.

Figure 2 shows the results of simulations at a moderate walking speed (*U*′ = 0.43). Animations of these simulations can be found in Supplementary Videos S1, S2. Regardless of whether a distributed mass or point-mass model is used, optimization of a Net COM Work cost function results in duty factors close to 1 (Figures 2A,B) with highly compliant leg actuation. Simultaneous contact results in a near-constant net vertical ground reaction force, reducing oscillation of the center of mass. In the distributed mass model, the forward bias of the center of mass results in lower peak forces in the hindlegs relative to the forelegs.

Minimizing Individual Limbs COM Work results in alternating periods of single-stance vaulting, with double-hump GRF profiles (Figures 2C,D). While peak forces do not change between the point mass and distributed mass models, the duty factor is extended in the forelimbs for the distributed mass case. This results in higher impulse for the forelimbs, as required to enable cyclical body pitching (Polet and Bertram, 2019).

Under a point mass model, the ILCW and Limb Extension Work are equivalent, and correspond to the solution in Figure 2C. However, LEW differs from ILCW under a distributed mass model (Figure 2F). The solution uses alternating periods of double and triple stance with double-hump ground reaction forces and duty factors close to 0.5– uniquely matching the empirical solution (Figure 2E) in these respects. The addition of a larger force rate penalty (matching Polet and Bertram, 2019) and more realistic forelimb and glenoacetabular lengths (from Bobbert et al., 2007) result in better empirical agreement, with smoother force peaks (Supplementary Figure S1; Supplementary Video S3).

### 3.2 Power Traces

#### 3.2.1 Solution Optimizing Net COM Work

**FIGURE 3:**
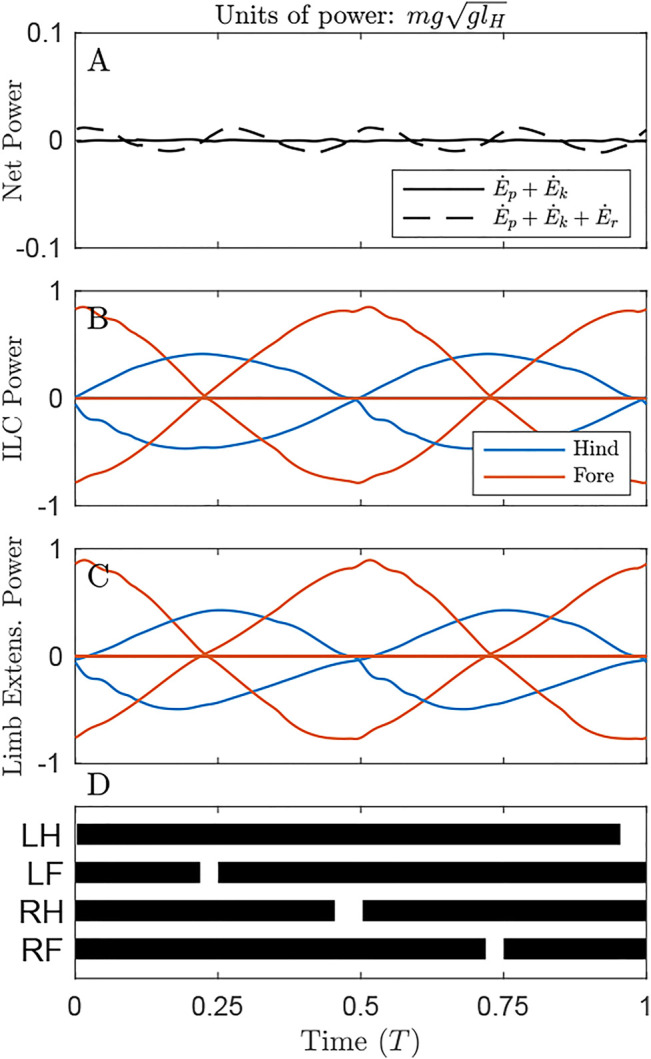
Power through time for the solution minimizing Net COM Work, under the distributed mass model, showing the origination of cost under different metrics. **(A)** Net power on the COM remains zero throughout the cycle (solid line), but system energy fluctuates slightly due to pitching of the body (dash line) **(B)** Individual Limbs COM Power and **(C)** Limb extension power are large, owing to substantial compression and extension of the limbs due to **(D)** large duty factors as shown in the gait diagram, leading to long periods of quadruple stance. Here and in the gait diagrams of Figures 4, 5, stance is plotted when GRF exceeds 0.02 body weights for a given limb.

**FIGURE 4:**
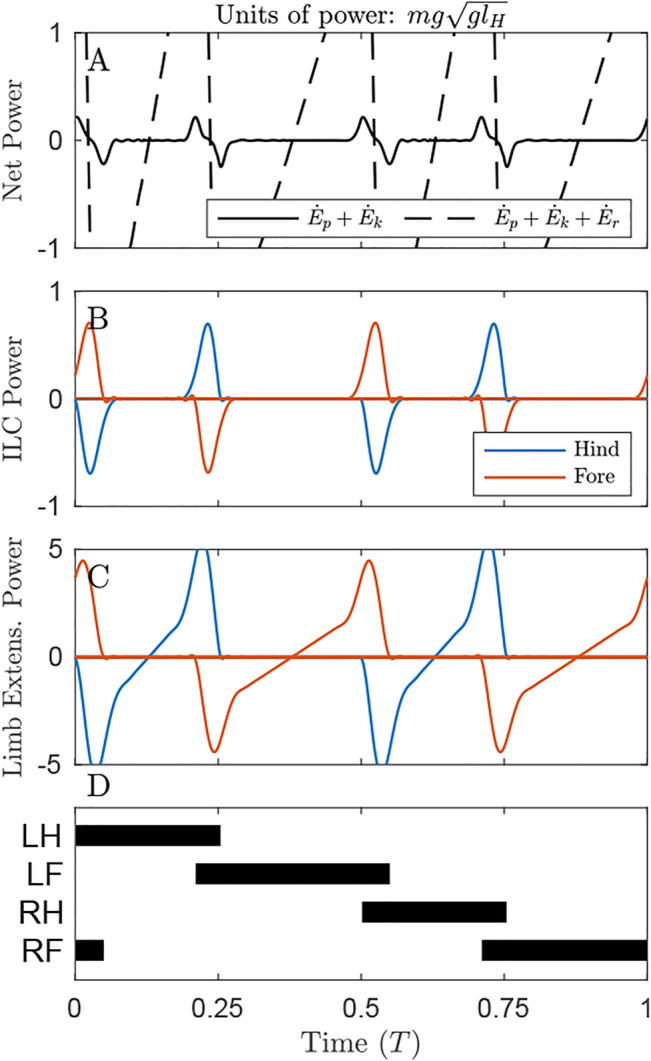
Power through time for the solution minimizing Individual Limbs COM Work under the distributed mass model. **(A)** There is a small oscillation of the Net COM Power (black line), but very large oscillations in the system energy changes (dash line) due to pronounced pitching (Supplementary Video S1). **(B)** Individual Limbs COM Power (ILCP) exhibits positive and negative peaks at transfer of support. Some power is inevitable due to redirecting the COM from a downward to upward trajectory. **(C)** The Limb Extension power is considerable, even during periods where ILCP is zero. Note that the *y*-axis here is 5 times that of Figures 3C, 5C. **(D)** The gait involves alternating periods of single and double stance.

**FIGURE 5:**
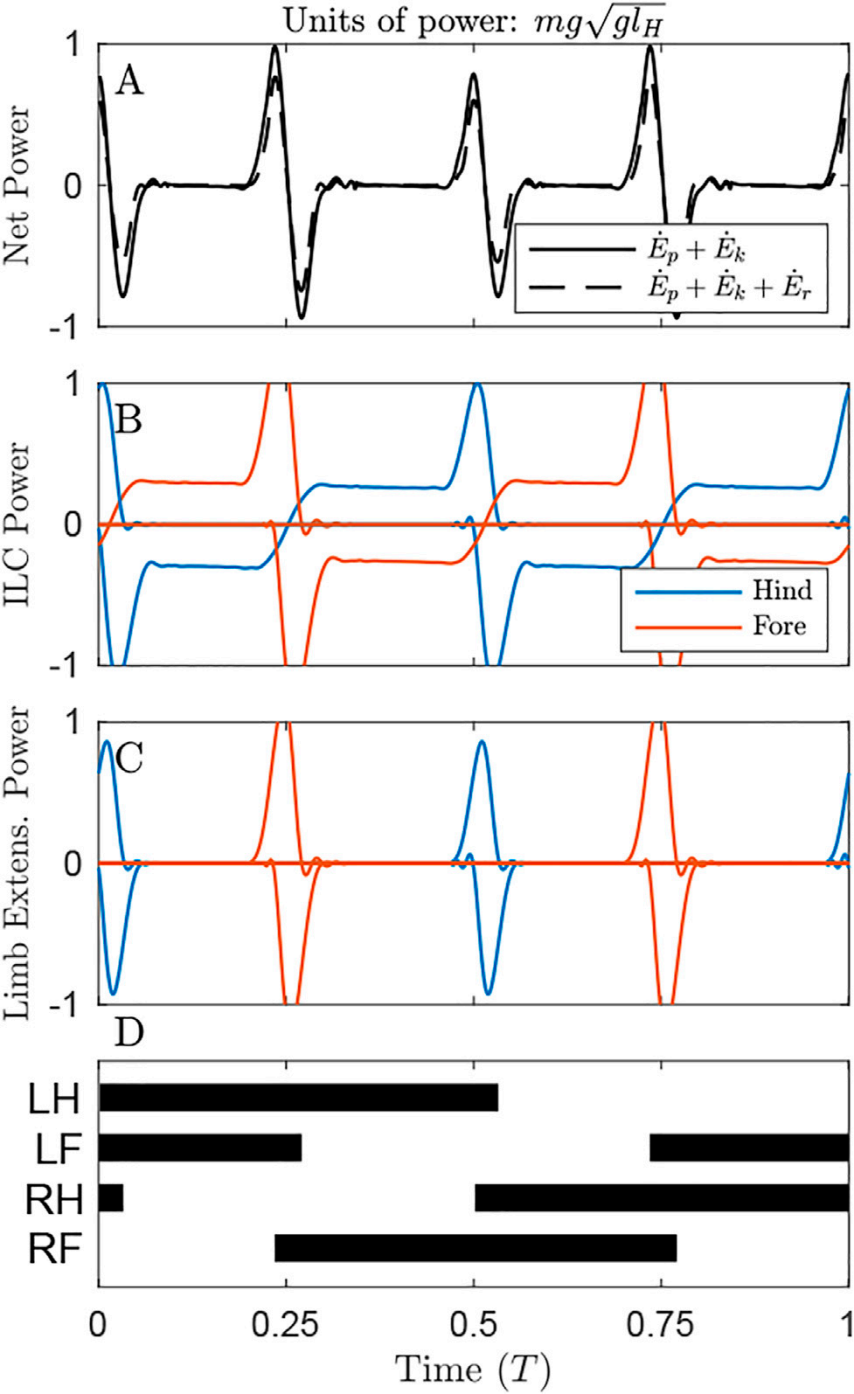
Power through time for the solution optimizing Limb Extension Work, under the distributed mass model. **(A)** Oscillations in Net COM Power (NCP, black line) and Net System Power (NSP, dash line) occur primarily at transfer of support. Accounting for changes in rotational energy results in a slight reduction of peak NSP compared to NCP at transfer of support. **(B)** Throughout the cycle, the gait exhibits continuous Individual Limbs COM Power, because the COM trajectory does not precisely follow the arc of each limb in stance. **(C)** Limb Extension Power exhibits peaks at transfer of support. These peaks are almost twice the value observed in Figure 3C, but the total positive work is lower since there are substantial periods of zero-cost passive vaulting. **(D)** The gait diagram shows a typical singlefoot walk with alternating periods of triple and double limb stance, with phase offset of 0.25. Note that left and right limbs can be swapped with no change to gait energetics in this planar model.

Power plots give a sense of how costly each solution is under the alternative cost functions. In Figure 3 (as in Figures 4, 5), only the optimal solution with the distributed mass model is shown for the given cost function.

By maintaining a virtually constant, vertical net ground reaction force equal to body weight (Supplementary Video S1), Net COM Power can be maintained at 0 for the duration of the cycle (Figure 3A, solid line). However, there are slight oscillations of total system power (dash line), as rotational kinetic energy varies throughout the cycle. This form of kinetic energy is ignored by NCW.

Despite exhibiting zero NCW, the solution minimizing this cost exhibits pronounced Individual Limbs COM Work (Figure 3B, net positive work 0.78 *mgl_H_*, Table 1), because limbs produce considerable forwards and backwards forces that provide simultaneous positive and negative power. Because little pitching is observed in this solution, the Limb Extension Power is almost equivalent to Individual Limb COM Power (Figure 3C).

**TABLE 1:** Positive work under various candidate cost functions, for simulations optimizing each cost function under a distributed mass model.

		Cost function optimized		
		NCW	ILCW	LEW
Total Positive Work (*mgl_H_*)	NCW	0.000	0.02	0.09
	NSW	0.002	0.51	0.04
	ILCW	0.78	0.09	0.41
	LEW	0.74	1.16	0.13
Percent Recovery		99.4	94	75

Optimizing NCW results in highly compliant gait, consistent with previous bipedal energetic models. A flat “Groucho walk” can maintain NCW of zero (Bertram et al., 2002; Kuo, 2007). However, a NCW cost function should be in some ways indeterminate. NCW of zero results in perfect exchange of COM Kinetic and Gravitational Potential Energies. An infinite number of trajectories could simulate this exchange, with the sufficient condition of mimicking a bead travelling on a frictionless surface between *x* = 0 and *x* = *D* in a set time (Figure 1C). Why then is the resulting gait completely flat? Here, the small force-rate penalty becomes the deciding factor. By using high duty factor with a flat gait, the force rate penalty is kept at a low value.

#### 3.2.2 Solution Optimizing Individual Limbs COM Work

When Individual Limbs COM Work is optimized, the Net COM Power fluctuates but remains small (Figure 4A, solid line), with total positive COM work of 0.02 *mgl_H_*. However, if changes in rotational dynamics are also considered, then the system exhibits enormous instantaneous changes in total energy (dash line), and total Net System Work of 0.51 *mgl_H_*.

This solution exhibits four peaks in Individual Limb COM power, corresponding to transfers of support (Figure 4B). A positive peak in one limb is met with a negative peak in the other; these largely cancel, resulting in low NCW (Figure 4A); similar to power curves in human walking (Donelan et al., 2002b). The majority of ILCW is performed during these periods of simultaneous positive and negative work.

This strategy consumes almost 9 times the positive actuator (limb extension) work as the strategy that minimizes Limb Extension Work (1.16 *mgl_H_* compared to 0.13 *mgl_H_*, Table 1). The ILCW strategy involves substantial pitching. While this pitching allows the COM to approximate single-stance vaulting over the fore and hind limbs sequentially, it results in a pronounced downward velocity of the hips or shoulders immediately prior to the moment of limb contact (Supplementary Video S2). This requires substantial negative work for the touchdown limb, and substantial positive work from the supporting limb (Figure 4C) to generate angular momentum redirecting the COM into a vaulting arc over the opposite limb.

#### 3.2.3 Solution Optimizing Limb Extension Work

The solution that optimizes Limb Extension Work (LEW) results in regular oscillations of COM energy (Figure 5A), with peaks corresponding to transfer of support and 4 times the net positive COM work as the ILCW solution (0.09 compared to 0.02 *mgl_H_*, Table 1). Accounting for changes in rotational kinetic energy (Figure 5A, dash line) results in a 55% reduction of the Net System Work (Table 1), due to a loss of rotational kinetic energy while translational kinetic energy increases (and *vice versa*). It is not clear, however, whether there is passive “transduction” between these forms (as in a ball rolling down a hill, Figure 1D), or whether it is a fully active process (determined by actuator work).

The solution that minimizes LEW exhibits substantial Individual Limbs COM Power throughout the entire cycle (Figure 5B). Peaks in power correspond to transfer of support, while flat regions of steady power correspond to portions of the passive vaulting phase. Power switches from negative in early stance to positive in late stance, after transfer of support has occurred on the opposite set of limbs and has redirected the center of mass velocity from downward to upward (Supplementary Video S1). The positive work performed, as measured with the Individual Limbs method, is 0.41 *mgl_H_*, more than four times the minimal value of 0.09 *mgl_H_* (Table 1; Figure 5B).

The optimal strategy for minimizing LEW exhibits prolonged passive phases of double-stance vaulting (Figure 5C), where the legs and torso act as a 4-bar linkage (Supplementary Video S2, Usherwood et al., 2007). These are punctuated by short phases of near-simultaneous positive and negative work, representing transfer of support. A supporting limb begins pushing, generating positive power, immediately prior to the touchdown limb generating negative power to absorb added energy and finish redirecting the hip or shoulder onto a vaulting path. The different heights of these power peaks are due to the mass bias. The forelimbs, being closer to the center of mass, exhibit higher peak power than the hindlimbs.

## 4 Discussion

Alternative metrics of work provide different insights into the energetics of locomotion. While these approximations are useful, they each implicitly ignore certain aspects of the dynamics and energetics of gait. Simplifying the system can be useful, and the metrics provide a way of quantifying gait and pointing to similarities between disparate organisms (Cavagna et al., 1977; Lee and Harris, 2018). It is tempting to point to a descriptive parameter as a prescriptive target of locomotion (or approximation of that target). Our tests on the prescriptive ability of these metrics leads to important insights about what they do– and do not– tell us about the determinants of organismal movement.

Net COM Work is the simplest metric of work to measure in practice. Changes in COM energy mean that something is performing work on it– most likely muscle tendon units. However, the converse is not necessarily true: observing no change in COM energy does not mean that something is not performing work on it. Simultaneous positive and negative work can be performed.

The solutions shown in Figures 2A,B and Figure 3 demonstrates the limitations of NCW. These solutions keep NCW at zero. Apparent fluctuations of potential and kinetic energy are exactly out of phase. Were such a gait observed in nature, an interpretation may be that it efficiently trades kinetic and potential energy of the center of mass [e.g. Griffin and Kram, 2000)]. However, the alternate metrics say something different. Both ILCW and LEW of this gait are high (Table 1), because it requires continuous simultaneous positive and negative work from individual limbs to manage the trajectory of the center of mass as if it were a bead on a wire, acting only under the influence of gravity (Figure 1C).

Like the mass on a track analogy, NCW does not account for rotational dynamics. If we calculate the Net System Work (NSW) for this solution (including changes in rotational kinetic energy), we see another way that NCW can overlook key system dynamics (Table 1). The NSW is 0.002 *mgl_H_* - still small, but appreciable compared to the zero NCW that the solution exhibits.

While the solutions optimizing NCW (Figures 2A,B) do not match a stereotypical singlefoot gait in force shape or duty factor (Figure 2E), they do match the gait in sequence of footfalls. The optimal solution does exhibit the stereotypical Hind-Fore-Hind-Fore contact pattern, with phase offset around 0.25. Notably, many primates and small mammals exhibit walking ground-reaction force patterns that are similar-single peaks with out-of-phase forelimb and hindlimb contacts, and a relatively compliant gait (Cartmill et al., 2002; Schmitt and Lemelin, 2002; d’Août et al., 2004; Schmidt, 2005; Webb et al., 2011). However, unlike the solution here, the NCW in these animals is appreciable, and percent recovery is often less than 50% (Ogihara et al., 2012; Demes and O’Neill, 2013). It therefore seems that, while these animals could keep NCW close to zero, their preferred gait is driven by other considerations.

There is another cost function implicit to these solutions that may be especially important at small sizes: muscle activation. Internal damping (Garcia et al., 2000; Weihmann, 2020), peak power (Hubel and Usherwood, 2015), and force generation (Kram and Taylor, 1990) have all been proposed as relatively costly for small animals. All these costs, while controversial in their physiological mechanisms, have a basis in more frequent muscle activation. In the present optimization framework, a force-rate penalty was imposed to regularize the solution, and has been linked with cost of muscle activation (van der Zee and Kuo, 2021). This penalty is likely responsible for the phase offset of 0.25 and the single-hump ground reaction forces in the minimal NCW solutions. Still other, non-energetic aspects may be as or more important determiners of locomotion at small sizes-for example, stability in arboreal habitats (Shapiro and Young, 2010).

Individual Limbs COM Work has been offered as an alternative to NCW that can capture simultaneous positive and negative work (Donelan et al., 2002a). However, it too is poorly prescriptive for quadrupedal walking. Whether or not rotational dynamics are included in the model, optimal ILCW calls for alternate periods of single stance while walking (Figures 2C,D). In the distributed mass model, this is achieved by extreme pitching, which results in extremely high actuator work (1.16 *mgl_H_*, Table 1) and NSW (0.51 *mgl_H_*), while the NCW is kept at a low value (0.02 *mgl_H_*).

While the present use of ILCW results in poor fidelity to walking in quadrupedal animals, Usherwood et al. (2007) and Usherwood and Self Davies (2017) used ILCW– with a distributed mass and point mass respectively– and achieved decent agreement with observed locomotion. This may be due to imposed constraints in the latter formulations. Usherwood et al. (2007) enforced periods of simultaneous hind and forelimb contact, while Usherwood and Self Davies (2017) constrained duty factor to match empirical data. Applying these constraints to our ILCW model would result in a more natural-looking gait, but would not– as Usherwood and Self Davies (2017) point out– explain why a given duty factor or simultaneous hind-fore contact are preferred.

Our results, from a less constrained optimization problem, show that optimizing ILCW cannot simultaneously explain the duty factors, phasing and ground reaction forces employed by walking quadrupedal animals. A key reason is the influence of pitch rotation, which ILCW ignores. In many natural gaits, including quadrupedal walking, the pitching energies appear small. However, Polet (2021) showed that pitching energies may be kept small because they would otherwise be expensive. While the individual limbs method may correspond closely to Limb Extension Work in most natural gaits, which do not exhibit much pitching, it does not explain why those gaits are non-pitching gaits.

Only the distributed mass model with optimal LEW resulted in a four-beat gait with duty factors close to 0.5 and double-hump GRF profiles (Figure 2F)– the stereotypical cursorial quadruped pattern. The pattern appears expensive both from the perspective of NCW and ILCW. It exhibits larger NCW than the other solutions (Table 1), and the percent recovery is relatively low at 75%– though this value more closely matches locomotion in dogs, who have percent recoveries between 50 and 70% in walking (Griffin et al., 2004). The ILCW is large (Figure 5B) even during passive stance phase with zero actuator power (Figure 5C), because the center of mass velocity is seldom oriented perpendicular to any leg in stance (Supplementary Video S1). It is only by considering the work of extending the leg that the natural, four-beat strategy becomes economical.

The pattern emerges as a tradeoff between cost of transfer of support–which favours more evenly distributed contacts (Ruina et al., 2005; Polet, 2021)– and cost of pitching the body–which favours simultaneous contact of the hind and forelimbs. For a Murphy number less than one– as occurs in dogs, horses, and likely most mammals– the four-beat singlefoot gait tends to be favoured (Polet, 2021).

The dynamics at transfer of support are, however, nontrivial. It is not immediately clear why, for example, even footfalls are optimal (even with a mass biased toward the forelimbs). We invoke three heuristics to explain why the four-beat singlefoot walk optimizes LEW:

1) Distributed contacts lowering collisional costs. As discussed by Ruina et al. (2005), contacts at regular intervals allow the contact velocity to be relatively consistent. As the cost of redirecting this velocity will be roughly proportional to its magnitude squared, keeping all contact velocities approximately equal lowers the total cost.
2) The pre-touchdown pushoff. Work-minimal bipedal walking benefits from pushing off with the support limb immediately prior to contact of the touchdown limb. The same strategy can be observed here, with a double-peak in the ground reaction force close to transfer of support (Figure 2F), and positive peak axial power coming immediately before negative peak power (Figure 5C). How the limb on the opposite end of the body might affect this transfer of support is not trivial, but the following effect is apparent (point three).
3) Work-free reaction at the opposite stance leg. Transfer of support causes a large peak force at one end of the body. When the radius of gyration is smaller than the moment arm– that is, the Murphy Number is less than one (Usherwood, 2020a; Polet, 2021)– the other end of the body will pitch downward. This downward pitching need not cost any extra work, however, as the opposite limb can simply increase its applied force to avoid changing length. This is observed as slight increases in force in midstance, at the moment the opposite pair of legs undergoes transfer of support (Figure 2F), consistent with the horse-inspired Murphy number of 0.82. When transfer of support at one of the hind legs occurs at midstance for the foreleg (and *vice versa*), the foreleg is vertical and in an optimal position to resist the applied force.

It is not immediately clear, however, how all these effects intermingle at the instant of contact. The interpretations above deserve more scrutiny under a formal collisional mechanical analysis.

### 4.1 Implications for Interpreting Biological Gait

It is often tempting to use simple metrics of biological locomotion to infer an underlying priority of the motor control system. The results here point to where the metrics might fail in the analysis of quadrupedal walking. A very widespread approach is to consider the changes in center of mass kinetic and potential energy as an identifier both of gait type and relative economy. Cavagna et al. (1977) noted that many walking animals– from primates, to birds, to dogs– exhibit out-of-phase kinetic and potential energy exchange during walking.

Since a point-mass pendulum exhibits similar out of phase potential and kinetic exchange, this kind of locomotion has been called “pendular” and the degree to which the total system energy remains constant is often parameterized as “pendular recovery” (Biknevicius et al., 2013; Shimada et al., 2017). The comparison has led to a conflation of out-of-phase kinetic and potential energy exchange– equivalently, low Net COM Work– and economical walking (Griffin and Kram, 2000; Reilly et al., 2007; Biknevicius et al., 2013; Shimada et al., 2017). Further, various authors have pointed to transduction of COM kinetic and gravitational potential energy as the primary mechanism allowing animals to walk with low energetic cost by reducing muscular work (Cavagna et al., 1977; Fischer and Lilhe, 2011; Pontzer et al., 2014; Bryce and Williams, 2017; Clayton and Hobbs, 2017).

The results of the present analysis challenge these ideas. Exact transduction of potential and kinetic energy is possible with the quadrupedal apparatus, but is rarely used in nature. Optimizing on NCW results in a solution that is unlike the passive vaulting gaits cited as “pendular” (Cavagna et al., 1977; Griffin et al., 2004; Fischer and Lilhe, 2011). Furthermore, tracking ${\dot{E}}_{P}+{\dot{E}}_{K}$ can lead to over- or underestimating the system energy changes (Table 1), as rotational energy changes are neglected.

Instead, we argue that the natural fluctuations in kinetic and potential COM energy are not the mechanism of quadrupedal walking, or even high walking economy, but a biproduct of optimizing a different cost function. The benefit of the “pendular strategy” is not really the passive transduction of kinetic energy into gravitational. Rather, it is the ability for the legs to remain straight, and the muscles to do little to no work, while the body translates forward (Tucker, 1975; Griffin et al., 2004). Indeed, the reduction of gravity (which ought to provide less opportunity for transduction between these modes) changes energetic cost little in bipedal walking and results in a slight reduction in cost (Farley and McMahon, 1992; Hasaneini et al., 2017). Quadrupeds can emulate perfect transduction of gravitational and kinetic energy; the apparent energy “savings” in this case are not savings at all, but require costly simultaneous positive and negative work.

Nevertheless, we believe that NCW– and its related parameterizations, pendular recovery and the “collision angle” (Lee et al., 2011)– can be usefully applied to gait analysis in two key ways. The first is as a gait classification scheme. Pendular recovery and collision angles have been used to identify subtle changes in gait within elephants (Ren and Hutchinson, 2008), birds (Usherwood et al., 2008), primates (Demes and O’Neill, 2013) and numerous other taxa (Lee and Harris, 2018). The second is to identify what portions of the stride could be most costly (Donelan et al., 2002b; Lee et al., 2011). As Figure 5 shows, the portions of stride corresponding to large fluctuations in Net COM Power also correspond to peaks in Limb Extension Power. However, the correspondence does not hold in all gaits (Figures 3, 4), and identifying costly portions of a stride does not mean that eliminating those portions would result in a reduction of cost (Kuo and Donelan, 2010; Simpson et al., 2019). As the number of limbs increases, it becomes harder to isolate the work of individual limbs, and NCW can be the only measurable form of work remaining (Zani et al., 2005; Lee et al., 2011). Our present results, however, promote caution in interpreting NCW as representative of energetic cost.

Individual Limbs COM Work is a tempting fix to NCW metrics. Simultaneous positive and negative work can be somewhat identified, while measuring the value *in vivo* remains practical even if more difficult (requiring, in principle, only limb-specific ground reaction forces and kinematic integration constants, e.g., average horizontal speed). It has been readily applied as an optimization paradigm (Alexander and Jayes, 1978; Usherwood and Self Davies, 2017), as it is simpler than computing axial limb work with associated rotational dynamics. However, the results here demonstrate that 1) it can be far removed from LEW (and muscle work: Sasaki et al., 2009) and that 2) its prescriptive solutions are not always biologically realistic (unless highly constrained: Usherwood et al., 2007; Usherwood and Self Davies, 2017).

None of the cost functions predicted gait well in a point mass model. Even though the most natural solution exhibits relatively small rotational energies compared to kinetic and potential energies of the center of mass (Figure 5A), rotational dynamics are a prerequisite to obtaining the solution. In this model, rotational dynamics are exploited to provide sequential passive phases of vaulting, while distributing contacts. Forces are applied work-free in the stance leg to accommodate transfer of support at the other end of the body. The model hints at other subtle ways to simultaneously reverse kinetic and rotational momentum at contact, but further analysis is required to understand the dynamics of this transition.

The combination of axial limb work with pitching dynamics in a planar model is more complex than the other cases considered here, and is difficult to measure, yet is still a useful simplification. This combination alone resulted in a four-beat walking gait, similar in many respects to the gaits employed by many quadrupedal mammals. The similarity between the optimal and natural gait could be interpreted as pointing to a biological mechanism. Specifically, it suggests that the four-beat vaulting gait is an emergent strategy from optimizing muscle work (per unit distance) during locomotion, and that axial limb work captures the dominant marginal cost for quadrupeds that use a vaulting walking gait.

However, the evidence provided here is indirect. By comparing metabolic expenditure to axial limb work empirically in different conditions, for example walking at a range of speeds, we would be able to test whether axial limb work is a good proxy for cost. Establishing muscle work or metabolic cost of transport as an optimization criterion of mammalian locomotion will require perturbation studies across many species in many conditions, similar to ongoing studies in human gait selection (Abram et al., 2019; Wong et al., 2019; Schroeder et al., 2021).

The models presented here are deliberately reductionist, and neglect several effects. Notably, lateral motions of the body are ignored, and these would be especially important for distinguishing symmetrical gaits that differ from a phase offset of 0.5 (i.e., Left-Left-Right-Right contact, vs*.* Left-Right-Left-Right). The gait diagram in Figure 5D, for example, is a diagonal sequence gait, while horses use a lateral sequence gait in walking. In the present model, there is no energetic difference between these gaits, and the optimizer in this case happened on the diagonal sequence by chance. However, the use of diagonal sequence gaits by some species, and lateral sequence by others, is a longstanding problem (Hildebrand, 1967) and the energetic consequences can only be resolved in 3D models (e.g., Usherwood and Self Davies, 2017).

The LEW results point to other areas where the model can be improved. LEW is an abstraction of joint work, which itself is an abstraction of muscle work. The cost of locomotion is not exactly proportional to axial limb work during locomotion. Ground reaction forces are not entirely leg-axial during quadrupedal walking in general (Jayes and Alexander, 1978; Usherwood, 2020b), and various antagonist muscles co-contract (Fischer and Lilhe, 2011). Isometric contraction and muscle (de)activation (Kushmerick and Paul, 1977; van der Zee and Kuo, 2021) are metabolically costly, and passive dissipation contributes to the work of locomotion (Zelik and Kuo, 2010). The model offers no explanation for why different mammals exhibit different limb phase relationships (Loscher, 2015), or why some mammals do not exhibit a double-hump ground reaction force profile (Webb et al., 2011; Demes and O’Neill, 2013). It also required stride length as an input, since there is low cost for frequent steps due to massless legs and low muscle (de)activation cost. Further refinement of the model presented here may provide clues as to which features of mammal morphology and physiology are responsible for their patterns of locomotion.

## 5 Conclusion

Simple metrics are used to quantify work in quadrupedal locomotion, and are often posited as determinants of the strategy employed. Here we tested the prescriptive ability of Net COM Work (NCW), Individual Limbs COM Work (ILCW) and Limb Extension Work (LEW) to predict the four-beat walking strategy typical of cursorial quadrupedal mammals. Optimizing NCW results in a highly compliant gait where the COM remains at a near constant height and velocity, while optimizing ILCW results in phases of single stance. Only optimizing LEW with distributed mass results in a gait that matches the stereotypical quadruped pattern.

Optimizing on NCW shows that perfect transduction of COM potential and kinetic energy are possible, but at the cost of extremely high limb work. While the compliant gait that emerges compares favourably to the gait of small mammals in footfall sequence and force shape, we believe this is due to the force-rate penalty imposed in our simulations for numerical regularization. This may have biological significance, as costs similar to force-rate likely become important as size decreases. ILCW has a poor correspondence to LEW in a distributed mass model, as the center of mass does not exactly follow the arcing trajectory of the limbs in passive stance during singlefoot walking. While NCW and ILCW serve as useful descriptive tools in gait analysis, they have limited prescriptive power. ILCW in particular has been used in predictive modelling frameworks with reasonable fidelity to natural gait. However, in a less constrained framework, as in the present study, the fidelity of its predictions are meagre.

Our results suggest that the stereotypical walking pattern in cursorial mammals does not optimally manage transduction of potential and kinetic energy, nor does it minimize the work that individual limbs do on the center of mass. Rather, it lowers the cost of positive muscle work by keeping LEW low. This is likely accomplished by a combination of 1) passive vaulting phases where the limbs remain straight and do no work (even if ILCW and NCW may be non-zero), interspersed with 2) distributed contacts minimizing translational collisional losses, assisted by 3) pre-footstrike pushoffs and 4) work free reaction at the limb in stance during transfer of support of the opposite pair, due to a low Murphy number.

## Data Availability

The general optimization code is available at https://doi.org/10.5281/zenodo.5593594. The main overhead scripts for this paper are included as Supplemental Data. The raw data supporting the conclusion of this article will be made available by the authors, without undue reservation.

## References

[B1] Abram S. J. Selinger J. C. Donelan J. M. (2019). Energy Optimization Is a Major Objective in the Real-Time Control of Step Width in Human Walking. J. Biomech. 91, 85–91. 10.1016/j.jbiomech.2019.05.010 31151794

[B2] Alexander R. M. Jayes A. S. (1978). Optimum Walking Techniques for Idealized Animals. J. Zoolog. 186, 61–81. 10.1111/j.1469-7998.1978.tb03357.x

[B3] Alexander R. M. (1980). Optimum Walking Techniques for Quadrupeds and Bipeds. J. Zoolog. 192, 97–117. 10.1111/j.1469-7998.1980.tb04222.x

[B4] Basu C. Wilson A. M. Hutchinson J. R. (2019). The Locomotor Kinematics and Ground Reaction Forces of Walking Giraffes. J. Exp. Biol. 222, jeb159277. 10.1242/jeb.159277 30510118

[B5] Bertram J. E. A. (2016). “Concepts in Locomotion,” in Understanding Mammalian Locomotion: Concepts and Applications (Hoboken, NJ: John Wiley & Sons), 111–141. 10.1002/9781119113713.ch5

[B6] Bertram J. E. A. D'antonio P. Pardo J. Lee D. V. (2002). Pace Length Effects in Human Walking: “Groucho” Gaits Revisited. J. Mot. Behav. 34, 309–318. 10.1080/00222890209601949 19260181

[B7] Betts J. T. (2010). “Practical Methods for Optimal Control and Estimation Using Nonlinear Programming,” in Advances in Design and Control (Philadelphia, Pennsylvania, USA: Society for Industrial and Applied Mathematics). 10.1137/1.9780898718577

[B8] Biknevicius A. R. Mullineaux D. R. Clayton H. M. (2004). Ground Reaction Forces and Limb Function in Tölting Icelandic Horses. Equine Vet. J. 36, 743–747. 10.2746/0425164044848190 15656508

[B9] Biknevicius A. R. Reilly S. M. McElroy E. J. Bennett M. B. (2013). Symmetrical Gaits and center of Mass Mechanics in Small-Bodied, Primitive Mammals. Zoology 116, 67–74. 10.1016/j.zool.2012.05.005 23195056

[B10] Bobbert M. F. Alvarez C. B. G. van Weeren P. R. Roepstorff L. Weishaupt M. A. (2007). Validation of Vertical Ground Reaction Forces on Individual Limbs Calculated from Kinematics of Horse Locomotion. J. Exp. Biol. 210, 1885–1896. 10.1242/jeb.02774 17515415

[B11] Bryce C. M. Williams T. M. (2017). Comparative Locomotor Costs of Domestic Dogs Reveal Energetic Economy of Wolf-like Breeds. J. Exp. Biol. 220, 312–321. 10.1242/jeb.144188 27811300

[B12] Buchner H. H. F. Savelberg H. H. C. M. Schamhardt H. C. Barneveld A. (1997). Inertial Properties of Dutch Warmblood Horses. J. Biomech. 30, 653–658. 10.1016/S0021-9290(97)00005-5 9165402

[B13] Cartmill M. Lemelin P. Schmitt D. (2002). Support Polygons and Symmetrical Gaits in Mammals. Zool. J. Linn. Soc. 136, 401–420. 10.1046/j.1096-3642.2002.00038.x

[B14] Cavagna G. A. Heglund N. C. Taylor C. R. (1977). Mechanical Work in Terrestrial Locomotion: Two Basic Mechanisms for Minimizing Energy Expenditure. Am. J. Physiology-Regulatory, Integr. Comp. Physiol. 233, R243–R261. 10.1152/ajpregu.1977.233.5.r243 411381

[B15] Clayton H. M. Hobbs S.-J. (2017). The Role of Biomechanical Analysis of Horse and Rider in Equitation Science. Appl. Anim. Behav. Sci. 190, 123–132. 10.1016/j.applanim.2017.02.011

[B16] D'Aout K. Vereecke E. Schoonaert K. De Clercq D. Van Elsacker L. Aerts P. (2004). Locomotion in Bonobos (*Pan paniscus*): Differences and Similarities between Bipedal and Quadrupedal Terrestrial Walking, and a Comparison with Other Locomotor Modes. J. Anat. 204, 353–361. 10.1111/j.0021-8782.2004.00292.x 15198700 PMC1571309

[B17] Demes B. O'Neill M. C. (2013). Ground Reaction Forces and center of Mass Mechanics of Bipedal Capuchin Monkeys: Implications for the Evolution of Human Bipedalism. Am. J. Phys. Anthropol. 150, 76–86. 10.1002/ajpa.22176 23124531

[B18] Donelan J. M. Kram R. Kuo A. D. (2002a). Mechanical Work for Step-to-step Transitions Is a Major Determinant of the Metabolic Cost of Human Walking. J. Exp. Biol. 205, 3717–3727. 10.1242/jeb.205.23.3717 12409498

[B19] Donelan J. M. Kram R. Kuo A. D. (2002b). Simultaneous Positive and Negative External Mechanical Work in Human Walking. J. Biomech. 35, 117–124. 10.1016/S0021-9290(01)00169-5 11747890

[B20] Farley C. T. McMahon T. A. (1992). Energetics of Walking and Running: Insights from Simulated Reduced-Gravity Experiments. J. Appl. Physiol. 73, 2709–2712. 10.1152/jappl.1992.73.6.2709 1490989

[B21] Farrell B. J. Bulgakova M. A. Beloozerova I. N. Sirota M. G. Prilutsky B. I. (2014). Body Stability and Muscle and Motor Cortex Activity during Walking with Wide Stance. J. Neurophysiol. 112, 504–524. 10.1152/jn.00064.2014 24790167 PMC4122701

[B22] Fischer M. S. Blickhan R. (2006). The Tri-segmented Limbs of Therian Mammals: Kinematics, Dynamics, and Self-Stabilization-A Review. J. Exp. Zool. 305A, 935–952. 10.1002/jez.a.333 17029268

[B23] Fischer M. S. Lilhe K. E. (2011). Dogs in Motion. Dortmund, Germany: VDH Service GmbH.

[B24] Fukuoka Y. Habu Y. Fukui T. (2015). A Simple Rule for Quadrupedal Gait Generation Determined by Leg Loading Feedback: A Modeling Study. Sci. Rep. 5, 8169. 10.1038/srep08169 25639661 PMC4313093

[B25] Full R. J. Koditschek D. E. (1999). Templates and Anchors: Neuromechanical Hypotheses of Legged Locomotion on Land. J. Exp. Biol. 202, 3325–3332. 10.1242/jeb.202.23.3325 10562515

[B26] Garcia M. Kuo A. Peattie A. Wang P. Full R. (2000). “Damping and Size: Insights and Biological Inspiration,” in Adaptive Motion of Animals and Machines, Montreal, Canada, 7.

[B27] Gill P. E. Murray W. Saunders M. A. (2005). SNOPT: An SQP Algorithm for Large-Scale Constrained Optimization. SIAM Rev. 47, 99–131. 10.1137/s0036144504446096

[B28] Gill P. E. Murray W. Saunders M. A. Wong E. (2015). User’s Guide for SNOPT 7.5: Software for Large-Scale Nonlinear Programming. San Diego, La Jolla, CA: Center for Computational Mathematics Report CCoM 15-1, Department of Mathematics, University of California.

[B29] Gray J. (1944). Studies in the Mechanics of the Tetrapod Skeleton. J. Exp. Biol. 20, 88–116. 10.1242/jeb.20.2.88 17601942

[B30] Griffin T. M. Kram R. (2000). Penguin Waddling Is Not Wasteful. Nature 408, 929. 10.1038/35050167 11140670

[B31] Griffin T. M. Main R. P. Farley C. T. (2004). Biomechanics of Quadrupedal Walking: How Do Four-Legged Animals Achieve Inverted Pendulum-like Movements? J. Exp. Biol. 207, 3545–3558. 10.1242/jeb.01177 15339951

[B32] Guertin P. A. (2012). Central Pattern Generator for Locomotion: Anatomical, Physiological, and Pathophysiological Considerations. Front. Neurol. 3, 183. 10.3389/fneur.2012.00183 23403923 PMC3567435

[B33] Hasaneini S. J. Schroeder R. T. Bertram J. E. A. Ruina A. (2017). The converse Effects of Speed and Gravity on the Energetics of Walking and Running. bioRxiv 2017, 201319. 10.1101/201319

[B34] Hildebrand M. (1976). “Analysis of Tetrapod Gaits: General Considerations and Symmetrical Gaits,” in Neural Control of Locomotion. Vol. 18 of Advances in Behavioral Biology. Editors Herman R. M. Grillner S. Stein P. Stuart D. (New York: Plenum Press New York), 203–236. 10.1007/978-1-4757-0964-3_9

[B35] Hildebrand M. (1967). Symmetrical Gaits of Primates. Am. J. Phys. Anthropol. 26, 119–130. 10.1002/ajpa.1330260203

[B36] Hoyt D. F. Taylor C. R. (1981). Gait and the Energetics of Locomotion in Horses. Nature 292, 239–240. 10.1038/292239a0

[B37] Hubel T. Y. Usherwood J. R. (2015). Children and Adults Minimise Activated Muscle Volume by Selecting Gait Parameters that Balance Gross Mechanical Power and Work Demands. J. Exp. Biol. 218, 2830–2839. 10.1242/jeb.122135 26400978 PMC4582168

[B38] Jayes A. S. Alexander R. M. (1978). Mechanics of Locomotion of Dogs (*Canis familiaris*) and Sheep (*Ovis aries*). J. Zoolog. 185, 289–308. 10.1111/j.1469-7998.1978.tb03334.x 700246

[B39] Kram R. Taylor C. R. (1990). Energetics of Running: A New Perspective. Nature 346, 265–267. 10.1038/346265a0 2374590

[B40] Kuo A. D. Donelan J. M. (2010). Dynamic Principles of Gait and Their Clinical Implications. Phys. Ther. 90, 157–174. 10.2522/ptj.20090125 20023002 PMC2816028

[B41] Kuo A. D. (2007). The Six Determinants of Gait and the Inverted Pendulum Analogy: A Dynamic Walking Perspective. Hum. Move. Sci. 26, 617–656. 10.1016/j.humov.2007.04.003 17617481

[B42] Kushmerick M. J. Paul R. J. (1977). Chemical Energetics in Repeated Contractions of Frog Sartorius Muscle at 0° C. J. Physiol. 267, 249–260. 10.1113/jphysiol.1977.sp011811 301567 PMC1283612

[B43] Lee D. V. Bertram J. E. A. Anttonen J. T. Ros I. G. Harris S. L. Biewener A. A. (2011). A Collisional Perspective on Quadrupedal Gait Dynamics. J. R. Soc. Interf. 8, 1480–1486. 10.1098/rsif.2011.0019 PMC3163420 21471189

[B44] Lee D. V. Harris S. L. (2018). Linking Gait Dynamics to Mechanical Cost of Legged Locomotion. Front. Robot. AI 5, 111. 10.3389/frobt.2018.00111 33500990 PMC7805771

[B45] Lee D. V. Isaacs M. R. Higgins T. E. Biewener A. A. McGowan C. P. (2014). Scaling of the Spring in the Leg during Bouncing Gaits of Mammals. Integr. Comp. Biol. 54, 1099–1108. 10.1093/icb/icu114 25305189 PMC4296203

[B46] Lee D. V. McGuigan M. P. Yoo E. H. Biewener A. A. (2008). Compliance, Actuation, and Work Characteristics of the Goat Foreleg and Hindleg during Level, Uphill, and Downhill Running. J. Appl. Physiol. 104, 130–141. 10.1152/japplphysiol.01090.2006 17947498 PMC2413412

[B47] Lee D. V. Todhunter R. J. Foels W. S. Jo Williams A. Lust G. Bertram J. E. A. (1997). Multiple Force Platform Analysis of the Canine Trot: A New Approach to Assessing Basic Characteristics of Locomotion. Vet. Comp. Orthop. Traumatol. 10, 160–169. 10.1055/s-0038-1632588

[B48] Loscher D. M. (2015). Kinematische Anpassungen zur Kollisionsreduktion im Schritt vierfüßiger Lauftiere. Berlin, Germany: Dr. rer. Nat., Freie Universität Berlin.

[B49] Manchester Z. Kuindersma S. (2017). “Variational Contact-Implicit Trajectory Optimization,” in International Symposium on Robotics Research (ISRR) (Puerto Varas, Chile: ISRR).

[B50] Ogihara N. Makishima H. Hirasaki E. Nakatsukasa M. (2012). Inefficient Use of Inverted Pendulum Mechanism during Quadrupedal Walking in the Japanese Macaque. Primates 53, 41–48. 10.1007/s10329-011-0265-3 21874286

[B51] Patterson M. A. Hager W. W. Rao A. V. (2015). A *ph* mesh Refinement Method for Optimal Control. Optim. Control. Appl. Meth. 36, 398–421. 10.1002/oca.2114

[B52] Patterson M. A. Rao A. V. (2014). GPOPS-II. ACM Trans. Math. Softw. 41, 1–37. 10.1145/2558904

[B53] Polet D. T. Bertram J. E. A. (2019). An Inelastic Quadrupedal Model Discovers Four-Beat Walking, Two-Beat Running, and Pseudo-elastic Actuation as Energetically Optimal. PLOS Comput. Biol. 15, e1007444. 10.1371/journal.pcbi.1007444 31751339 PMC6871776

[B55] Polet D. T. (2021). The Murphy Number: How Pitch Moment of Inertia Dictates Quadrupedal Walking and Running Energetics. J. Exp. Biol. 224, jeb228296. 10.1242/jeb.228296 33462135

[B56] Pontzer H. Raichlen D. A. Rodman P. S. (2014). Bipedal and Quadrupedal Locomotion in Chimpanzees. J. Hum. Evol. 66, 64–82. 10.1016/j.jhevol.2013.10.002 24315239

[B57] Reilly S. M. McElroy E. J. Biknevicius A. R. (2007). Posture, Gait and the Ecological Relevance of Locomotor Costs and Energy-Saving Mechanisms in Tetrapods. Zoology 110, 271–289. 10.1016/j.zool.2007.01.003 17482802

[B58] Ren L. Hutchinson J. R. (2008). The Three-Dimensional Locomotor Dynamics of African (*Loxodonta africana*) and Asian (*Elephas maximus*) Elephants Reveal a Smooth Gait Transition at Moderate Speed. J. R. Soc. Interf. 5, 195–211. 10.1098/rsif.2007.1095 PMC2705974 17594960

[B59] Riddick R. C. Kuo A. D. (2020). Mechanical Work Accounts for Most of the Energetic Cost in Human Running. bioRxiv 2020, 2020. 10.1101/2020.09.22.309161 PMC8755824 35022431

[B60] Rohatgi A. (2019). WebPlotDigitizer. Available at: https://automeris.io/WebPlotDigitizer .

[B61] Ruina A. Bertram J. E. A. (2003). “Problems with Some Historical Locomotion Energy Accounting,” in Proceedings of the 27th Meeting of the American Society of Biomechanics, Toledo, OH.

[B62] Ruina A. Bertram J. E. A. Srinivasan M. (2005). A Collisional Model of the Energetic Cost of Support Work Qualitatively Explains Leg Sequencing in Walking and Galloping, Pseudo-elastic Leg Behavior in Running and the Walk-To-Run Transition. J. Theor. Biol. 237, 170–192. 10.1016/j.jtbi.2005.04.004 15961114

[B63] Sasaki K. Neptune R. R. Kautz S. A. (2009). The Relationships between Muscle, External, Internal and Joint Mechanical Work during normal Walking. J. Exp. Biol. 212, 738–744. 10.1242/jeb.023267 19218526 PMC2726854

[B64] Schmidt M. (2005). Quadrupedal Locomotion in Squirrel Monkeys (Cebidae:*Saimiri sciureus*): A Cineradiographic Study of Limb Kinematics and Related Substrate Reaction Forces. Am. J. Phys. Anthropol. 128, 359–370. 10.1002/ajpa.20089 15838834

[B65] Schmitt D. Lemelin P. (2002). Origins of Primate Locomotion: Gait Mechanics of the Woolly Opossum. Am. J. Phys. Anthropol. 118, 231–238. 10.1002/ajpa.10048 12115279

[B66] Schroeder R. T. Croft J. L. Bertram J. E. A. (2021). Evaluating the Energetics of Entrainment in a Human-Machine Coupled Oscillator System. Sci. Rep. 11, 15804. 10.1038/s41598-021-95047-x 34349146 PMC8338938

[B67] Shapiro L. J. Young J. W. (2010). Is Primate-like Quadrupedalism Necessary for fine-branch Locomotion? A Test Using Sugar Gliders (*Petaurus breviceps*). J. Hum. Evol. 58, 309–319. 10.1016/j.jhevol.2009.12.002 20153016

[B68] Shimada H. Kanai R. Kondo T. Yoshino-Saito K. Uchida A. Nakamura M. (2017). Three-dimensional Kinematic and Kinetic Analysis of Quadrupedal Walking in the Common Marmoset (*Callithrix jacchus*). Neurosci. Res. 125, 11–20. 10.1016/j.neures.2017.06.005 28711711

[B69] Simpson C. S. Welker C. G. Uhlrich S. D. Sketch S. M. Jackson R. W. Delp S. L. (2019). Connecting the Legs with a spring Improves Human Running Economy. J. Exp. Biol. 222, jeb202895. 10.1242/jeb.202895 31395676 PMC6765174

[B70] Tucker V. A. (1975). The Energetic Cost of Moving about. Am. Sci. 63, 413–419. 1137237

[B71] Usherwood J. R. (2022). Legs as Linkages: An Alternative Paradigm for the Role of Tendons and Isometric Muscles in Facilitating Economical Gait. J. Exp. Biol. 225, jeb243254. 10.1242/jeb.243254 35258605 PMC8987730

[B72] Usherwood J. R. (2020a). An Extension to the Collisional Model of the Energetic Cost of Support Qualitatively Explains Trotting and the Trot-Canter Transition. J. Exp. Zool. 333, 9–19. 10.1002/jez.2268 PMC6916616 31033243

[B73] Usherwood J. R. Self Davies Z. T. (2017). Work Minimization Accounts for Footfall Phasing in Slow Quadrupedal Gaits. eLife 6, 630–650. 10.7554/eLife.29495 PMC5599235 28910262

[B74] Usherwood J. R. Szymanek K. L. Daley M. A. (2008). Compass Gait Mechanics Account for Top Walking Speeds in Ducks and Humans. J. Exp. Biol. 211, 3744–3749. 10.1242/jeb.023416 19011215 PMC2978950

[B75] Usherwood J. R. (2020b). The Possibility of Zero Limb-Work Gaits in Sprawled and Parasagittal Quadrupeds: Insights from Linkages of the Industrial Revolution. Integr. Org. Biol. 2, obaa017. 10.1093/iob/obaa017 33073170 PMC7545857

[B76] Usherwood J. R. Williams S. B. Wilson A. M. (2007). Mechanics of Dog Walking Compared with a Passive, Stiff-Limbed, 4-bar Linkage Model, and Their Collisional Implications. J. Exp. Biol. 210, 533–540. 10.1242/jeb.02647 17234623

[B77] van der Zee T. J. Kuo A. D. (2021). The High Energetic Cost of Rapid Force Development in Muscle. J. Exp. Biol. 224, jeb233965. 10.1242/jeb.233965 33707194

[B78] van der Zee T. J. Lemaire K. K. van Soest A. J. (2019). The Metabolic Cost of *In Vivo* Constant Muscle Force Production at Zero Net Mechanical Work. J. Exp. Biol. 222, jeb199158. 10.1242/jeb.199158 30877229

[B79] Webb A. A. Kerr B. Neville T. Ngan S. Assem H. (2011). Kinematics and Ground Reaction Force Determination: A Demonstration Quantifying Locomotor Abilities of Young Adult, Middle-Aged, and Geriatric Rats. JoVE 2011, 2138. 10.3791/2138 PMC3059563 21403621

[B80] Weihmann T. (2020). Survey of Biomechanical Aspects of Arthropod Terrestrialisation - Substrate Bound Legged Locomotion. Arthropod Struct. Develop. 59, 100983. 10.1016/j.asd.2020.100983 33160205

[B81] Wong J. D. Selinger J. C. Donelan J. M. (2019). Is Natural Variability in Gait Sufficient to Initiate Spontaneous Energy Optimization in Human Walking? J. Neurophysiol. 121, 1848–1855. 10.1152/jn.00417.2018 30864867 PMC6589705

[B82] Zani P. A. Gottschall J. S. Kram R. (2005). Giant Galaápagos Tortoises Walk without Inverted Pendulum Mechanical-Energy Exchange. J. Exp. Biol. 208, 1489–1494. 10.1242/jeb.01554 15802673

[B83] Zelik K. E. Kuo A. D. (2010). Human Walking Isn't All Hard Work: Evidence of Soft Tissue Contributions to Energy Dissipation and Return. J. Exp. Biol. 213, 4257–4264. 10.1242/jeb.044297 21113007 PMC2992466

