# Supplementary material for: Competing Models of Work in Quadrupedal Walking: Center of Mass Work is Insufficient to Explain Stereotypical Gait
Source: Front Bioeng Biotechnol. 2022 May 12;10:826336. doi: 10.3389/fbioe.2022.826336 (PMC9135023; doi:10.3389/fbioe.2022.826336)
Supplement: Supplementary file 2 [file Presentation1.PDF]

Supplementary Material

1 SUPPLEMENTARY VIDEO CAPTIONS

Video S1. Animations of optimal solutions displayed in Figure 2 of the main manuscript. Here the net ground reaction force (GRF) from all limbs ($\mathbf{F}_{\mathrm{net}}$) is shown as a black arrow originating at the center of mass (COM), while the COM velocity ($\mathbf{V}_{\mathrm{COM}}$) is a grey arrow. When these arrows are 90° apart, Net COM Work (NCW) is zero, and when they have components parallel, NCW is non-zero. GRF of individual limbs are represented by the opacity of the limbs, with forces acting along the axis of each leg. When $\mathbf{V}_{\mathrm{COM}}$ is 90° to a leg, the Individual Limbs COM Work (ILCW) of that leg is zero, while $\mathbf{V}_{\mathrm{COM}}$ aligned with an active leg leads to finite ILCW from that leg. Circles represent radii of gyration from the COM, and red and blue are forelimbs and hindlimbs respectively.

Video S2. Animations of optimal solutions displayed in Figure 2 of the main manuscript. Here the velocity of the shoulder and hips are shown as a grey arrows. Ground reaction forces of individual limbs are represented by the opacity of the limbs, with forces acting along the axis of each leg. When a leg is oriented 90° to the velocity of its attachment point (hips or shoulders), the Limb Extension Work (LEW) of that leg is zero. When an active leg has a component aligned with the attachment velocity, the LEW is non-zero. Circles represent radii of gyration from the COM, and red and blue are forelimbs and hindlimbs respectively.

Video S3. Animations of the optimal solution from a more realistic horse model, with empirical glenoacetabular distance and forelimb length, and a realistic force rate penalty matching Polet and Bertram (2019). In the first half of the video, the net ground reaction force (GRF) from all limbs ($\mathbf{F}_{\mathrm{net}}$) is shown as a black arrow originating at the center of mass (COM), while the COM velocity ($\mathbf{V}_{\mathrm{COM}}$) is a grey arrow. In the second half, velocity of the shoulder and hips are shown as a grey arrows. Ground reaction forces of individual limbs are represented by the opacity of the limbs, with forces acting along the axis of each leg. Circles represent radii of gyration from the COM, and red and blue are forelimbs and hindlimbs respectively.

2 SUPPLEMENTARY FIGURES

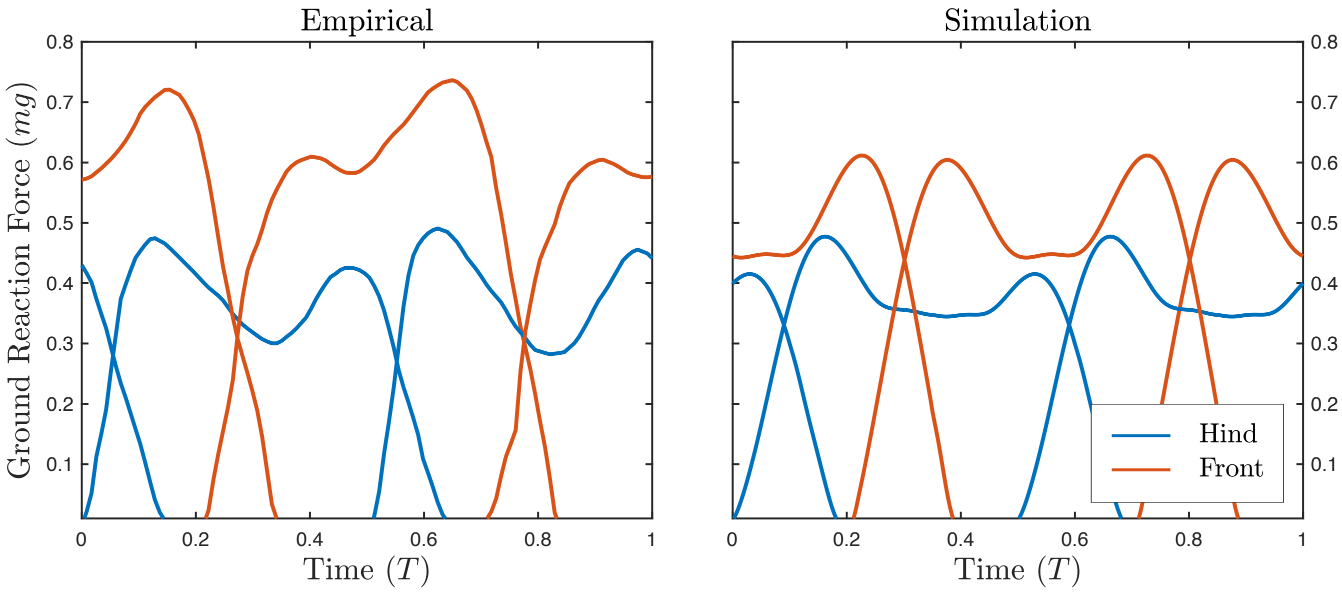

Figure S1. Empirical ground reaction forces for a horse (left) compared to solution minimizing limb extension work (right) with leg lengths and glenoacetabular distance matching proportions in Bobbert *et al.* (2007) and a force-rate penalty matching Polet and Bertram (2019).
